# Predictors of COVID-19 vaccination status among youth in eThekwini, South Africa: results from the AYAZAZI RIGHTS study

**DOI:** 10.1080/16549716.2026.2737555

**Published:** 2026-10-06

**Authors:** Janan J. Dietrich, Tatiana E. Pakhomova, C. Andrew Basham, Kalysha Closson, Bongiwe Zulu, Julie Jesson, Campion Zharima, Mags Beksinska, Rishav Singh, Erica Dong, Angela Kaida

**Affiliations:** a African Social Sciences Unit of Research and Evaluation (ASSURE), A Division of the Wits Health Consortium, Johannesburg, South Africa; b Perinatal HIV Research Unit (PHRU), Faculty of Health Sciences, University of the Witwatersrand, Johannesburg, South Africa; c Health Systems Research Unit, South African Medical Research Council, Bellville, South Africa; d Faculty of Health Sciences, Simon Fraser University, Burnaby, BC, Canada; e Wits MRU (MatCH Research Unit), Department of Obstetrics and Gynaecology, Faculty of Health Sciences, University of the Witwatersrand, Durban, South Africa; f Center for Epidemiology and Research in Population Health (CERPOP), Inserm, Université de Toulouse, Toulouse, France; g Centre for Health Policy, School of Public Health, University of the Witwatersrand, Johannesburg, South Africa; h BC Children’s Hospital Research Foundation, Vancouver, BC, Canada; i Women’s Health Research Institute, University of British Columbia, Vancouver, BC, Canada

**Keywords:** Vaccines, vaccine hesitancy, young adults, SARS-CoV-2, South Africa

## Abstract

**Background:**

Understanding which factors impact vaccine uptake is important for supporting ongoing and future vaccine programmes among youth.

**Objective:**

This study examined predictors of COVID-19 vaccination uptake among South African youth in the eThekwini District.

**Methods:**

Cross-sectional data from an online survey (AYAZAZI RIGHTS: December 2021–May 2022) of youth aged 18–24 in the eThekwini district, South Africa, were analyzed using a gender-stratified logistic regression of vaccination status on candidate predictors of interest (k = 14). A full model with backwards selection using Akaike’s Information Criterion was employed to remove predictors that did not add to the model’s explanatory power.

**Results:**

Of 1383 participants, 54% were women, with a median sample age of 21.8 years. Overall, 37.9% (*n* = 525) of participants had received a COVID-19 vaccine, 58.6% of whom were women. In this cross‑sectional, observational analysis, the most consistent predictors of vaccination status were older age, living with seniors in the home, and personal proximity to severe pandemic outcomes, specifically knowing people who had been hospitalized or died due to COVID-19. Gender‑specific predictors included ethnicity, having children for women, employment, and relationship status.

**Conclusion:**

Socio-demographic as well as community factors predicted receiving a COVID-19 vaccination, varying by gender. Better understanding of modifiable factors to support vaccine uptake, and curbing vaccine hesitancy related to mistrust and misinformation, will help support South Africa’s immunization programs among youth, and can help inform targeted, inclusive, and equity-oriented public-health strategies.

## Paper Context

- **Main findings**: Just under 38% of South African youth in our study had been vaccinated against coronavirus disease 2019 (COVID-19), with predictors of COVID-19 vaccination for both men and women including socio-demographic and community-related factors, such as measures of household makeup and knowing people impacted by COVID-19.
- **Added knowledge**: Evidence from this study suggests gendered differences both for vaccination uptake and for vaccination hesitation.
- **Global health impact for policy and action**: The findings highlight the need for a better understanding of modifiable factors to support vaccine uptake, furthermore, curbing vaccine hesitancy related to mistrust and misinformation will be central to supporting South Africa’s immunization programs among youth.

## Background

The COVID-19 pandemic was a significant global health crisis, with vaccines being central to reducing the morbidity and mortality of COVID-19. The COVID-19 pandemic saw one of the fastest vaccine development programs in history, with efforts coordinated across the globe [1,2]. Once effective and safe vaccines were developed, wide-spread vaccination campaigns began [3], and while over 70% of the world population has received at least one COVID-19 vaccination [4], both between-country [5] and within-country [6] disparities in vaccination coverage and access have been reported, with the lowest coverage reported across Africa [4]. Importantly, disparities in vaccination coverage may reflect multiple and overlapping factors, including vaccine hesitancy, limited access to vaccination services, poor vaccine availability and other structural barriers. Thus, lower vaccination coverage should not be associated with greater vaccine hesitancy.

South Africa reported the largest number of COVID-19 cases among any African nation to date [7,8]. This is despite the swift implementation of a nation-wide lockdown initiated in March 2020, to slow the spread of COVID-19 [9]. In early 2021, South Africa announced a robust vaccine rollout plan; vaccines were provided free-of-charge through the national government roll-out program, beginning vaccinations for health care workers in February 2021, followed by older adults in May 2021, then young adults under 35 were granted access in July 2021, with 12–17-year-olds in October 2021 [10]. Challenges such as a lack of timely access to vaccines, delays in distribution and limited outreach to younger populations contributed to COVID-19 resurgence, subsequent continued lockdowns, and low country-wide vaccination coverage [11,12]. These challenges further illustrate how vaccination uptake is influenced not only by individuals’ willingness to vaccinate but also by other structural factors such as limited access to services and vaccine availability. By the end of 2023, approximately 39 million vaccine doses had been distributed in South Africa, equating to approximately 49.5% of the total population being either fully or partially vaccinated, inclusive of 18 million fully vaccinated adults over the age of 18 (46%) [13].

Despite increased availability of vaccines and evidence supporting vaccine effectiveness [14,15], hesitation to vaccinate against COVID-19 remained high among South African youth, likely contributing to lower vaccination rates, where just under 41% of 18–35-year-olds were vaccinated by mid-2023 [13], with disparities across both gender and ethnic groups [16]. South African studies examining vaccination intentions among adults, including healthcare workers, have found vaccine hesitancy, which refers to the delay in acceptance or refusal of vaccination despite the availability of vaccination services [17], to be associated with younger age, low income, low education, being single, being less informed (about COVID-19), and perceived low risk [18,19], as well as religious and cultural beliefs [20]. Concerns about side effects, limited access to the online vaccine registration, distrust of government, belief in conspiracy theories, and peer influence were associated with hesitancy to vaccinate among South Africans [18,21], including in qualitative studies with young people [22]. In analyzing these disparities, it is critical to conceptually distinguish vaccine hesitancy from vaccine uptake, which is the final behavioral action shaped by the intersection of individual willingness and structural systems.

In 2022, approximately 44% of South Africa’s population was below the age of 25, with adolescents and young adults between the ages of 15–25 making up 16% of the total population [23]. Given that many South Africans also live in multi-generational homes that include older adults [24], young people likely play an important role in the COVID-19 response because of their influence on vaccination behaviour among household members and their impact on COVID-19 mortality and morbidity [25,26]. Understanding which factors impact COVID-19 vaccine uptake is important to support both COVID-19 efforts and other immunization campaigns, as well as future vaccine programmes including current HIV vaccine research ongoing in South Africa [27,28]. Yet few studies have investigated the predictors contributing to low levels of vaccination among this priority population.

In this study, we aimed to 1) characterize sociodemographic and pandemic-related characteristics of vaccinated and unvaccinated urban-dwelling youth in KwaZulu-Natal, South Africa; 2) develop a prediction model to examine the variables that best predict vaccination status among individuals who received a government-approved vaccination as part of the government program; and 3) summarize factors related to vaccine hesitancy among youth who were not vaccinated against COVID-19.

## Methods and materials

### Study population and setting

Our study population consisted of adolescents and young adults living in eThekwini district, Durban, South Africa, the third largest municipality in the country [29]. Located on South Africa’s east coast in the KwaZulu-Natal (KZN) Province, eThekwini accounts for just over a third of the population of KZN, with high rates of poverty and unemployment reported among residents in the 2016 South African Community Survey [29]. Eligibility criteria for the study included residence in eThekwini district, Durban, South Africa, being between 16–24 years of age, having access to the internet through a mobile device or computer, and the ability to read in English and/or isiZulu.

### Study design and data collection

We analyzed cross-sectional data from the AYAZAZI RIGHTS (Rapid Investigation of Gendered Health Outcomes in the Time of SARS-CoV-2) survey, conducted December 2021–May 2022. Participants were recruited via the research and recruitment division of the Maternal and Child Health (MatCH) Research Unit, a clinical and behavioural research facility at the Witwatersrand University focused on sexual and reproductive health outcomes [30] in the eThekwini District, through community-based and youth-led organisations, inclusive of both the MatCH adult Community Advisory Board (CAB) and Adolescent CAB (aCAB). Recruitment was conducted through flyer distribution and word of mouth; the survey was additionally advertised online (via social media).

Participants completed an anonymous, self-administered online survey, available in English and/or isiZulu, via a web-link through a partnership with #datafree Moya Messenger App. The survey was created using REDCap (Research Electronic Data Capture) to examine experiences of COVID-19, including public health measures, sexual and reproductive health, and sociodemographics among youth residing in the eThekwini district. The questionnaire was pilot tested with community members and MaTCH research personnel and staff. The consent form stated confidentiality and that participants could omit answering questions they felt uncomfortable with. Additional study details have been published elsewhere [31].

### Analytic sample

Altogether, 2694 people accessed the survey online; those who did not consent (*n* = 303) or did not complete the survey (*n* = 142) were excluded from the study. Inclusion criteria were being 18 years or older and having completed the survey in >4.5 min; based on piloting, the research team determined that completion in under 4.5 min would be insufficient to adequately read and understand the questionnaire. Exclusion criteria included receipt of only one AstraZeneca vaccination (*n* = 207) and not having complete data for one or more variables of interest (*n* = 150). The sample was restricted to those ≥ 18 years to accurately capture those eligible for COVID-19 vaccines during December 2021–May 2022, and account for potential delays or other factors affecting accessibility [10–12]. AstraZeneca was briefly provided in South Africa in early 2021 as part of a limited, clinical trial evaluating effectiveness against circulating COVID-variants [32,33], but was halted due to ineffectiveness against common COVID-19 strains present in South Africa at that time [34]. Therefore, the AstraZeneca vaccine was never made available to the general public as an approved vaccine in South Africa. Due to low numbers, non-binary and gender non-conforming individuals were excluded from the final gender-stratified prediction analyses; they were included only in overall, bivariate analyses.

### Measures

#### Outcome

Our outcome, COVID-19 vaccination status, was measured with the survey questions ‘*Did you receive a COVID-19 vaccination?’* and *‘what kind of COVID-19 vaccination did you receive?’* Fully vaccinated individuals had received two doses of either Pfizer or Moderna or one dose of Johnson & Johnson (J&J). Individuals who received one dose of Pfizer or Moderna, or one dose of AstraZeneca and another vaccine, were classified as partially vaccinated. Both partially and fully vaccinated individuals were included as ‘vaccinated’ in the prediction models, in order to reflect the fact that vaccination campaigns were ongoing at the start of study data collection, and not all individuals may have been offered, eligible, or had yet taken a second dose by the start of the study.

Individuals who received only one AstraZeneca vaccination were excluded from the analytic sample. AstraZeneca was provided in South Africa in early 2021 as part of a phase III, clinical trial evaluating effectiveness against circulating COVID-variants before the trial was halted due to evidence of ineffectiveness against common COVID-19 strains present in South Africa at the time [32–34]; it was never made available to the general public as an approved vaccine in South Africa.

#### Candidate predictors

We tested 14 candidate predictors selected *a priori* from the current literature as well as theoretically plausible relationships. Studies have identified age as a significant predictor of vaccination status, with younger age associated with greater hesitancy and lower vaccination rates in both global [19,35], and South African studies [18,36]. Other frequently cited factors include education level, income, ethnicity, and marital/relationship status [35,37], with South African studies specifically reporting lower monthly income, education level, and relationship status (being single), as well as ethnicity, as significant predictors of lower vaccination acceptance and higher hesitancy [18,19]. Household composition as well as personal experiences with COVID-19 are also associated with vaccination behaviour, with South African studies finding that higher vaccine uptake [38] and lower hesitancy [36] were associated with living with someone who was vaccinated against COVID-19, as well as knowing someone who has had COVID-19 [36]. Global data indicate lower COVID-19 vaccination uptake among migrants and ethnic minorities, as a result of higher hesitancy, and access barriers [39].

Candidate socio-demographic predictors included: age in years (continuous; centered at the mean), where change in the outcome was parameterized as a one-year unit increase in age away from the mean value of 21.76 years for women and 21.88 years for men), race/ethnicity (Black [reference category]; coloured [40], in South Africa, the term Coloured refers to ‘any person of “mixed blood” as well as descendants from Black–White, Black–Asian, White–Asian, and Black–Coloured unions’; Indian/Asian; white), gender (men [reference category]; women; non-binary/gender non-conforming), being employed or in school (no [reference category]; yes), relationship status (single [reference category]; cohabitating; not-cohabitating), having children (no [reference category]; yes), cohabitating; non-cohabitating), sexual orientation (heterosexual/straight [reference category]; lesbian, gay, bisexual, queer and more [LGBQ+]), HIV status (negative [reference category]; positive; unknown or prefer not to say), and current monthly income (no income-R1600 [reference category]; R1601+). Two measures for household composition were included: number of seniors in the household (none; one; more than two), and number of children in the household (none; one to two; three or more), derived from the survey question ‘*Besides you, how many people currently live in your household*’; participants entered values for each of the three categories provided (“*number of adults aged 18–59 years; number of seniors 60 years; number of children 0–17 years*).

COVID-19 specific variables included the number of individuals known to the participant who were hospitalized during COVID-19 (none [reference category]; one to five; more than five) and the number of people who died from COVID-19 (none [reference category]; one to five; more than five). These were derived from the survey questions: 1) ‘*How many people do you personally know who were hospitalized because of COVID-19*?’ and 2) ‘*How many people do you personally know who died from COVID-19?*’, with response categories of ‘none’, ‘one to five people’, or ‘five or more people’. We also included experiences of xenophobia since the start of the COVID-19 pandemic (no [reference category]; yes), created via the survey question ‘*Since the start of the COVID-19 Pandemic, I have experienced Xenophobia*’, with possible answers yes vs. no.

#### Estimating vaccine hesitancy among unvaccinated youth

Participants, who were eligible to be vaccinated but had not yet received any government-approved COVID-19 vaccination (*n* = 735) were asked how likely they were to receive a vaccine. Participants answering anything other than ‘very likely’ were subsequently asked to provide up to three reasons for not receiving a COVID-19 vaccine, from a set of 11 pre-determined answers, which included an open-text box. Among those who did not receive a COVID-19 vaccination, we provided a descriptive overview of the aforementioned reasons identified by participants as salient to their decision not to vaccinate.

### Statistical analysis

Descriptive statistics, stratified by gender, summarized sociodemographic, health and behavioural characteristics of study participants. Predictor variables were assessed using single, self-reported survey questions. We assessed collinearity by examining variance inflation factors (VIF), for collinear variables with over-lapping explanatory power (VIF > 5). For objectives 1 and 2, bivariate (unadjusted) logistic regression models were used to estimate the associations between vaccination status (vaccinated) and candidate predictors, presented as odds ratios (OR), with confidence intervals (CI). Gender‑stratified multivariable logistic regression estimated the predicted probability of vaccination status by the candidate predictors. For model selection, we ran both a full model, then a secondary model using backwards selection with Akaike’s Information Criterion (AIC), k = 2, to remove candidate predictors that did not add to the model’s explanatory power and to identify a more parsimonious set of predictors while retaining the variables that contributed more substantially to the model fit. We used this stepwise approach to identify the most parsimonious variables among the 14 exploratory candidates, as youth-specific predictors of vaccination had not been previously established. As the missingness for our individual variables of interest was exceptionally low (under 3% for any single variable), we performed a complete‑case analysis. Out of 1,533 eligible participants, a total of 150 (9.8%) had missing data on either the outcome of interest or candidate predictors and were excluded from the analytic sample, yielding 1,383 participants with complete data (Figure 1). Model performance was assessed using pseudo-R-squared for prediction error, and the Hosmer–Lemeshow test, which measured the calibration of the logistic model by comparing observed and predicted events. The concordance statistic (c-statistic) was used to assess discrimination in logistic regression models [41]. In line with South African health research priorities [42], we performed gender-stratified analyses to investigate gendered differences in COVID-19 vaccination uptake.

**Figure 1.**
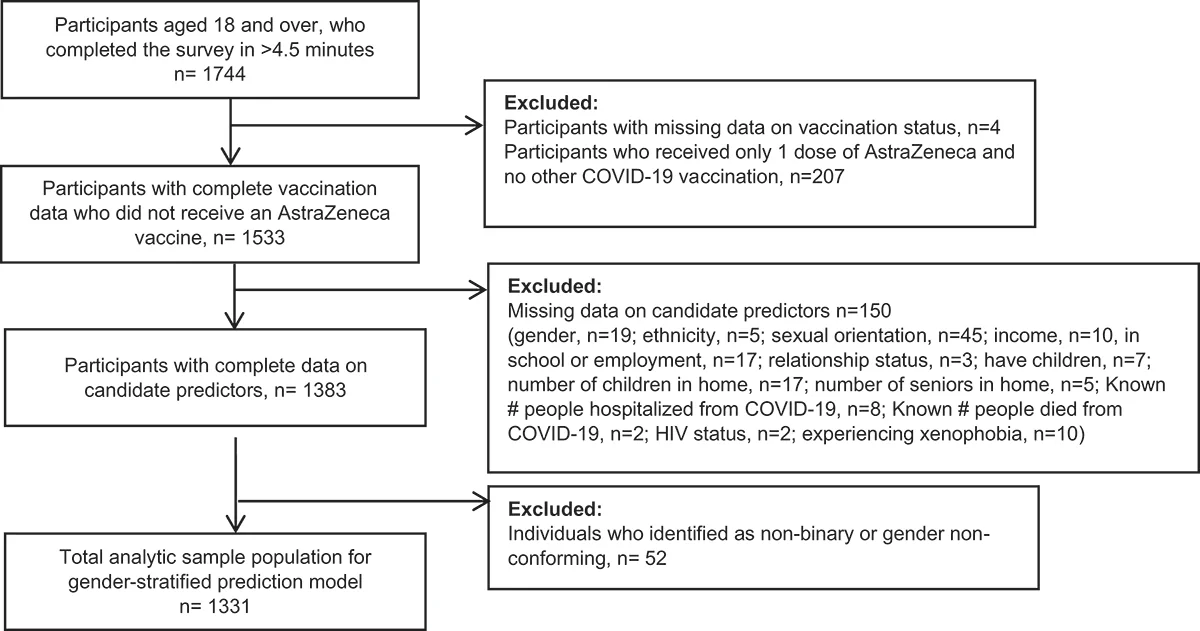
Analytic sample flow chart.

#### Sensitivity analyses

In sensitivity analyses, we included interaction terms for relationships of interest and used backwards selection with AIC to improve the parsimony of the model. Subsequent significant relationships that were retained were examined independently as separate (full) models. Studies have found that household composition, which includes multi-generational living situations where seniors and older adults live with younger people, impacts COVID-19 mortality and morbidity, as well as vaccination behaviour among household members [25,26]. Given the commonality of multigenerational homes in South Africa [24], we hypothesized that the number of seniors in the home may moderate the relationship between age and vaccination status. Furthermore, we hypothesized that migrant status and xenophobia [43] would have a negative impact on vaccination status, and as such wanted to better understand how socio-economic factors such as income and student status may influence this relationship.

## Results

### Participant characteristics

Of 1383 participants, 54% were women, 14.7% identified as lesbian, gay, bisexual, queer and more (LGBQ+), with a median age of 21.8 years. Fifty‑eight percent reported earning a monthly income of R1,600 or less (Table 1), and 27.6% reported that they were employed at the time of the study. Over a quarter reported knowing more than five individuals who had been hospitalized due to COVID-19 (25.6%), and 30.4% of participants reported knowing more than five individuals who had died due to COVID-19. Overall, 37.9% of participants had received ≥ 1 dose of a government‑approved COVID-19 vaccine. Men as well as non-binary individuals in our sample were more likely to be unvaccinated, while women were more likely to be vaccinated than not.

**Table 1.** Bivariate parameter estimates for vaccine status, participants ≥ 18 (*n* = 1383).

Candidate predictor	Overall, n = 1383	Vaccinated, n = 524 (37.9%)	Unvaccinated, n = 859 (62.1%)	OR (95% CI)	c-stat
Age, years (mean (SD))	21.83 (2.07)	22.53 (1.69)	21.41 (2.16)	1.34 (1.26–1.42)*	0.652
Gender (*N* (%))					
Boy/Man	585 (42.3)	206 (39.3)	379 (44.1)	REF	0.542
Girl/Woman	746 (53.9)	307 (58.6)	439 (51.1)	1.29 (1.03–1.61)*	
Non-binary	52 (3.8)	11 (2.1)	41 (4.8)	0.49 (0.24–0.95)*	
Race/Ethnicity (*N* (%))					
Black	1035 (74.8)	365 (69.7)	670 (78.0)	REF	0.570
Coloured	155 (11.2)	47 (9.0)	108 (12.6)	0.80 (0.55–1.14)	
Indian/Asian	135 (9.8)	83 (15.8)	52 (6.1)	2.93 (2.03–4.26)*	
White	58 (4.2)	29 (5.5)	29 (3.4)	1.84 (1.08–3.13)*	
Sexual Orientation (*N* (%))					
Heterosexual	1179 (85.2)	439 (83.8)	740 (86.1)	REF	0.512
LGBQ+	204 (14.8)	85 (16.2)	119 (13.9)	1.20 (0.89–1.63)	
Current student (*N* (%))					
No	786 (56.8)	329 (62.8)	457 (53.2)	REF	0.548
Yes	597 (43.2)	195 (37.2)	402 (46.8)	0.67 (0.54–0.84)*	
Employed (*N* (%))					
No	1001 (72.4)	307 (58.6)	694 (80.8)	REF	0.611
Yes	382 (27.6)	217 (41.4)	165 (19.2)	2.97 (2.33–3.79)*	
Monthly Income (*N* (%))					
R0 – R1600	807 (58.4)	238 (45.4)	569 (66.2)	REF	0.604
>R1600	576 (41.6)	286 (54.6)	290 (33.8)	2.36 (1.89–2.95)	
Relationship Status (*N* (%))					
Single	227 (16.4)	88 (16.8)	139 (16.2)	REF	0.549
Cohabitating	132 (9.5)	76 (14.5)	56 (6.5)	2.14 (1.39–3.33)*	
Not-cohabitating	1024 (74.0)	360 (68.7)	664 (77.3)	0.86 (0.65–1.15)	
Have children (*N* (%))					
No	810 (58.6)	270 (51.5)	540 (62.9)	REF	0.557
Yes	573 (41.4)	254 (48.5)	319 (37.1)	1.59 (1.28–1.99)*	
HIV status (*N* (%))					
negative	842 (60.9)	304 (58.0)	538 (62.6)	REF	0.525
positive	90 (6.5)	40 (7.6)	50 (5.8)	1.42 (0.91–2.19)	
unknown or PNTA	451 (32.6)	180 (34.4)	271 (31.5)	1.18 (0.93–1.49)	
Number of children in household (*N* (%))					
none	142 (10.3)	35 (6.7)	107 (12.5)	REF	0.562
1–2	656 (47.4)	234 (44.7)	422 (49.1)	1.70 (1.13–2.59)*	
3 or more	585 (42.3)	255 (48.7)	330 (38.4)	2.36 (1.57–3.62)*	
Number of seniors in household (*N* (%))					
none	474 (34.3)	131 (25.0)	343 (39.9)	REF	0.619
one	644 (46.6)	235 (44.8)	409 (47.6)	1.50 (1.16–1.95)*	
2 or more	265 (19.2)	158 (30.2)	107 (12.5)	3.87 (2.82–5.32)*	
Known people hospitalized from COVID-19 (*N* (%))					
none	125 (9.0)	37 (7.1)	88 (10.2)	REF	0.659
one to five	905 (65.4)	251 (47.9)	654 (76.1)	0.91 (0.61–1.39)	
more than five	353 (25.5)	236 (45.0)	117 (13.6)	4.80 (3.10–7.54)*	
Known people who died from COVID-19 (*N* (%))					
none	323 (23.4)	60 (11.5)	263 (30.6)	REF	0.712
one to five	638 (46.1)	180 (34.4)	458 (53.3)	1.72 (1.25–2.41)*	
more than five	422 (30.5)	284 (54.2)	138 (16.1)	9.02 (6.42–12.84)*	
Experienced xenophobia since start of Pandemic (*N* (%))					
No	1262 (91.3)	442 (84.4)	820 (95.5)	REF	0.555
Yes	121 (8.7)	82 (15.6)	39 (4.5)	3.90 (2.64–5.86)*	

Note: SE: standard error; m(SD): mean and standard deviation; c-stat: concordance statistic; COVID-19: Coronavirus disease 2019; LGBQ+: lesbian, gay, bisexual, queer and more; HIV: Human Immunodeficiency virus; PNTA: prefer not to answer; REF: reference category; OR: odds ratio; CI: confidence interval.

*p < 0.05.

Of the total, 328 were fully vaccinated (23.7% of the analytic sample), and 196 were partially vaccinated (14.2%) (Supplementary: Table A). At the time of the study, 39.7% (*n* = 543) of participants reported having ever had a confirmed COVID-19 infection. Participants were also asked about their perceptions of the importance of COVID-19 vaccination, with 633 (45.7%) overall answering that they believed vaccination to be very or extremely important for community health, and 617 (44.6%) reporting that they believed it to be very or extremely important for their own health. Among those who were unvaccinated (*n* = 859), only 82 (5.9% of the analytic sample) reported that they were very likely to get vaccinated in the future.

### Modeling predictors of vaccine status

#### Bivariate analyses

In bivariate analyses (Table 1), significant predictors of having at least one dose of a COVID-19 vaccine included age (OR 1.34; 95% CI 1.26–1.42), where for each year older than the average age in our sample, the probability of being vaccinated increased, being of white (OR 1.84; 95% CI 1.08–3.13) or Indian/Asian (OR 2.93; 95% CI 2.03–4.26) ethnicity, being employed at the time of the study (OR 2.97; 95% CI 2.33–3.79), being in a relationship and living with a romantic partner (OR 2.14; 95% CI 1.39–3.33), having children (OR 1.59; 95% CI 1.28–1.99), earning more than R1,600 per month (OR 2.36; 95% CI 1.89–2.95), knowing more than five people who had been hospitalized (OR 4.80; 95% 3.10–7.54), knowing anyone who had died due to COVID-19 (OR 1.72; 95% 1.25–2.41), as well as having experienced xenophobia since the start of the pandemic (OR 3.90; 95% CI 2.64–5.86) (Table 1). Those who were students at the time of the study (OR 0.67; 95% CI 0.54–0.84), and who identified as non-binary or gender non-conforming had a significantly lower likelihood of being vaccinated (OR 0.49; 95% CI 0.24–0.95).

#### Gender-stratified multivariate analyses

In gender‑stratified multivariate analyses (Table 2), we found 10 significant predictors of receiving at least one dose of a COVID-19 vaccination, across both full and reduced models. For men and women, both the full and reduced models discriminated well between those who were vaccinated and those who were not, with c-statistic scores all falling within the good-to‑excellent ranges (0.819, 8.22 for women; 0.886, 0.884 for men). While the Hosmer–Lemeshow test suggested that the reduced models fit the data better for both men (*p* = 0.517) and women (*p* = 0.967), all *p*-values were > 0.05, indicating good fit. Pseudo‑R^2^ values indicated that the models explained a moderate to modest amount of variance in vaccination status for both groups.

**Table 2.** Factors predicting vaccination status among AYAZAZI participants ≥ 18, by gender (*n* = 1331).

		Women (n = 746)				Men (n = 585)			
Predictor		Model 1 (full)		Model 2 (Backward AIC)		Model 1 (full)		Model 2 (Backward AIC)	
		aOR	95% CI	aOR	95% CI	aOR	95% CI	aOR	95% CI
Intercept		0.07	0.02–0.22	0.09	0.03–0.22	0.07	0.02–0.25	0.09	0.03–0.27
Centered age (mean)		1.31	1.17–1.46*	1.32	1.19–1.46*	1.28	1.11–1.47*	1.28	1.12–1.47*
Ethnicity	Black	REF				REF		REF	
	Coloured	1.10	0.62–1.93			0.48	0.21–1.11	0.52	0.23–1.18
	Indian/Asian	1.45	0.79–2.64			2.53	1.11–5.75*	2.23	1.01–4.97*
	white	0.82	0.34–1.97			12.45	3.79–40.88*	11.74	3.69–37.35*
Sexual Orientation	heterosexual	REF		REF		REF		REF	
	LGBQ+	2.40	1.38–4.18*	2.41	1.39–4.18*	2.55	1.16–5.59*	2.44	1.14–5.25*
Currently a student	no	REF				REF			
	yes	0.98	0.65–1.45			0.91	0.53–1.57		
Currently Employed	no	REF				REF			
	yes	1.44	0.85–2.45	1.66	1.10–2.52*	2.21	1.05–4.62*	2.38	1.40–4.03*
Relationship Status	single	REF		REF		REF		REF	
	cohabitating	2.06	0.99–4.29	2.03	0.99–4.18	0.79	0.27–2.33	0.95	0.34–2.65
	not cohabitating	0.63	0.37–1.06	0.63	0.38–1.06	0.37	0.19–0.73	0.38	0.19–0.75*
Have children	no	REF				REF			
	yes	0.67	0.45–1.01	0.67	0.46–0.99*	1.23	0.71–2.14		
Income level	R0-R1600	REF				REF			
	R1601+	1.23	0.77–1.98			1.02	0.52–1.99		
Number of children in household	none	REF				REF			
	one to two	1.20	0.56–2.57			2.01	0.85–4.76		
	three or more	1.40	0.62–3.15			1.71	0.70–4.20		
Number of seniors in household	none	REF		REF		REF		REF	
	one	2.28	1.45–3.66*	2.42	1.58–3.72*	1.16	0.65–2.07	1.33	0.77–2.27
	two or more	3.30	1.84–6.01*	3.70	2.15–6.35*	4.19	2.06–8.53*	4.65	2.41–9.05*
Known people hospitalized due to COVID-19	none	REF		REF		REF		REF	
	one to five	1.17	0.56–2.41	1.16	0.57–2.37	0.75	0.34–1.67	0.85	0.39–1.84
	five or more	3.40	1.52–7.58*	3.34	1.52–7.37*	2.52	1.05–6.08*	2.74	1.16–6.62*
Known people died due to COVID-19	none	REF		REF		REF		REF	
	one to five	1.97	1.14–3.41*	2.01	1.17–3.47*	2.13	1.11–4.08*	2.25	1.18–4.29*
	five or more	5.11	2.77–9.53*	5.42	2.92–10.04*	12.26	5.70–26.34*	12.62	5.96–26.69*
HIV Status	negative	REF				REF			
	positive	0.89	0.48–1.65			0.70	0.21–2.34		
	unknown/PTNA	0.91	0.60–1.40			0.83	0.51–1.36		
Experienced xenophobia since start of	no	REF		REF		REF		REF	
Pandemic	yes	3.67	1.76–7.65*	3.42	1.67–6.98*	6.30	2.84–13.95*	6.26	2.85–13.76*
Model performance									
Observations (n)		746		746		585		585	
R-squared		0.309		0.316		0.458		0.451	
c-statistic		0.819		0.822		0.886		0.884	
Hos-Lemeshow test (*p*-value)		0.188		0.517		0.193		0.967	

Note: aOR: adjusted odds ratio, 95% CI: 95% confidence interval; m(SD): mean and standard deviation; c-stat: concordance statistic; COVID-19: Coronavirus disease 2019; LGBQ+: lesbian, gay, bisexual, queer, and more; HIV: Human Immunodeficiency virus; PNTA: prefer not to answer; REF: reference category; AIC: Akaike’s Information Criterion.

*p < 0.05.

For women, significant predictors included age (adjusted OR 1.31; 95% CI 1.17–1.46), where each year older than the average age in our sample increased the probability of being vaccinated identifying as LGBQ+ (aOR 2.40; 95% CI 1.38–4.18), being employed – though only in the reduced model (aOR 1.66; 95% CI 1.10–2.52), and having experienced xenophobia since the start of the pandemic (aOR 3.67; 95% CI 1.76–7.65). Additionally, having seniors in the home (aOR 2.28; 95% CI 1.45–3.66) was associated with a higher likelihood of having a COVID-19 vaccine, a likelihood that increased when two or more seniors resided in the household (aOR 3.30; 95% CI 1.84–6.01). Knowing more than five people who had been hospitalized (aOR 3.40, 95% CI 1.52–7.58) or someone who had died due to COVID-19 (aOR 1.97; 95% CI 1.14–3.41) strongly predicted being vaccinated, with the strongest predictor being having known five or more people who had died due to COVID-19 (aOR 5.11; 95% CI 2.77–9.53). For women, having children was associated with a lower likelihood of vaccination (aOR 0.67; 95% CI 0.46–0.99), in the reduced model (Table 2).

For men, significant predictors included age, where every year increase from the mean age was associated with a greater likelihood of vaccination (aOR 1.28; 95% CI 1.11–1.47), ethnicity – being of Indian/Asian ethnicity [aOR 2.53; 95% CI 1.11–5.75] or white ethnicity [aOR 11.74]; however, the results were very imprecise, including in the reduced model (95% CI 3.69–37.35). Interpreting the effect size for white ethnicity requires caution, as the large adjusted odds ratio and wide confidence interval reflect substantial imprecision and statistical instability, which are driven by the very small subgroup sample size of white men in our stratified sample. Other predictors included being employed (aOR 2.21; 95% CI 1.05–4.62), identifying as LGBQ+ (aOR 2.55; 95% CI 1.16–5.59), having two or more seniors in the home (aOR 4.19; 95% CI 2.06–8.53), knowing five or more people who were hospitalized due to COVID-19 (aOR 2.52; 95% CI 1.05–6.08), and experiencing xenophobia since the start of the pandemic (aOR 6.30; 95% CI 2.84–13.95). Knowing someone who has died due to COVID-19 (aOR 2.13; 95% CI 1.11–4.08) also predicted being vaccinated, as did knowing five or more people who had died due to COVID-19 (aOR 12.26; 95% CI 5.70–26.34), but with no precision given the wide confidence intervals, which indicate uncertainty in the results. For men, being in a relationship but not living together was associated with a decreased likelihood of vaccination (aOR 0.37; 95% CI 0.19–0.73) (Table 2).

### Results overview

In bivariate analyses, we found that overall, study participants were more likely to be vaccinated if they were older, white or Indian/Asian, employed, living with a partner, had children, earned more than R1,600/month, had seniors living in the home, had personally known people hospitalized or who died due to COVID, or had experienced xenophobia since the start of the pandemic. Gender‑stratified regression analyses revealed slightly differing results, where for both men and women vaccination was predicted by age (being older), sexual orientation (identifying as LGBQ+), being employed (although only in the reduced model for women), having seniors living at home, experiencing xenophobia, and personally knowing people hospitalized or who died from COVID. For men, ethnicity was predictive of vaccination, while being in a relationship was associated with lower odds of vaccination; having children was linked to a lower chance of being vaccinated for women.

### *Sensitivity analyses*: interaction models

In exploratory moderation analyses, results for men indicated a significant relationship for income and experiencing xenophobia during COVID-19, where having a lower income and experiencing xenophobia was associated with an increased likelihood of vaccination (aOR 23.20; 95% CI 6.26–86.03), albeit with no precision. Similarly, we found a significant association between being a student and experiencing xenophobia, where not being enrolled in school and experiencing xenophobia was associated with a significantly increased likelihood of being vaccinated against COVID-19 (aOR 16.58; 95% CI 5.38–51.13), though also with no precision. Given the wide confidence intervals indicating substantial uncertainty and instability, we emphasize that interpretation of these results with caution (Supplementary: Table B). We also found significant interactions between age and number of seniors in the home, where for every year of increase in age, the impact of having one or more seniors in the home increased the likelihood of COVID-19 vaccination for women (aOR 1.35; 95% CI 1.10–1.64) and having two or more seniors in the home increased the likelihood for men (aOR 1.55; 95% CI 1.20–2.01).

#### Reasons for vaccine hesitancy among unvaccinated participants not interested in receiving a COVID-19 vaccination

Among the 742 participants (55%), who indicated they were unvaccinated and not interested in receiving a vaccine, 53.1% (*n* = 394) were women. Overall, the top reasons given for not receiving a COVID-19 vaccine was 1) not thinking that the vaccine was not safe (52.2%, *n* = 387), followed by 2) fear of vaccine side-effects (50.3%, *n* = 373), and 3) respondents thinking that the vaccine was not effective (40.8% *n* = 303) (Figure 2). More men compared with women indicated that they thought the vaccine was ineffective (47.4% vs. 35.0%), while women were more likely (57% vs. 42.5%) to report that they were concerned about side-effects. Few participants indicated that they did not think COVID-19 was a serious disease (*n* = 20) or reported difficulty with accessing a vaccine/difficulty getting to a vaccination centre (*n* = 21).

**Figure 2.**
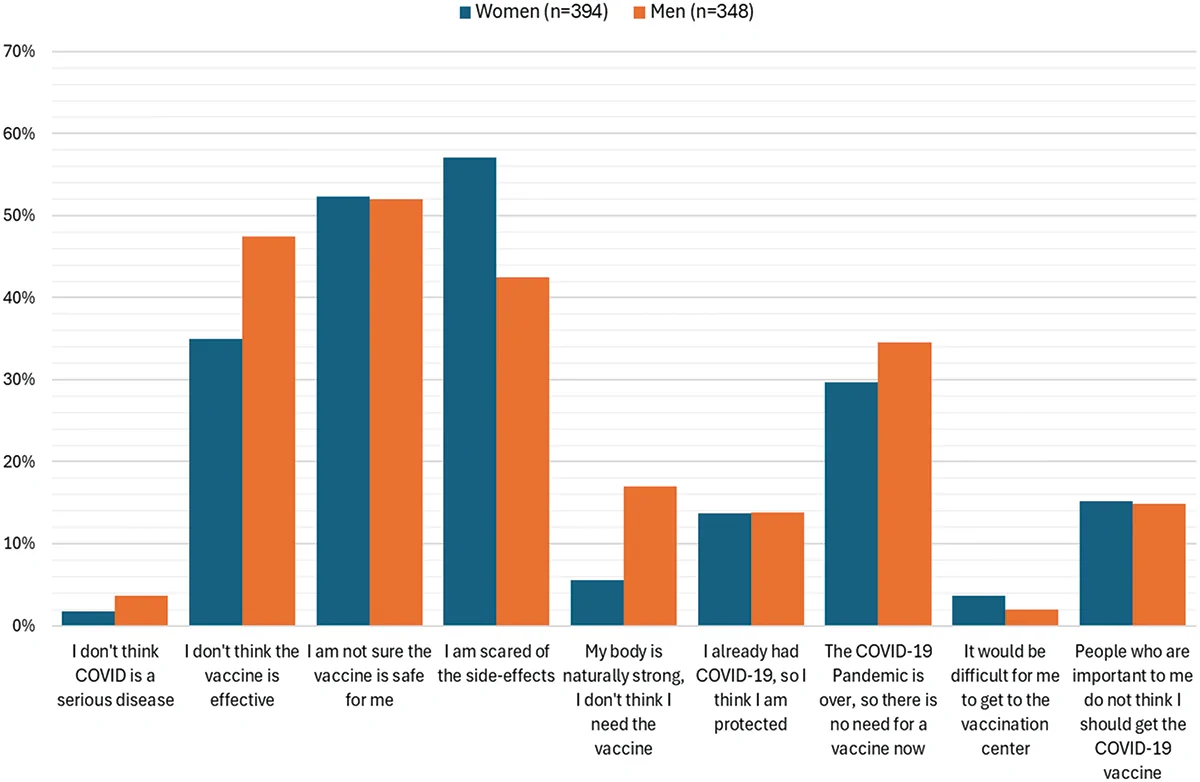
Top three reasons for not getting a COVID-19 vaccination, among unvaccinated men and women aged ≥ 18 (*n* = 742).

## Discussion

In this cross-sectional study, predictors of COVID-19 vaccination for both men and women spanned socio-demographic and community-related factors. Utilizing a gender-stratified approach, we found that vaccine‑uptake predictors reflected both shared factors, including age, sexual orientation, measures of household makeup, and knowing people who were impacted by COVID-19 (knowing people who had died, or hospitalized due to COVID-19), as well gender-specific factors, including relationship status, employment and ethnicity. In our study, just under 38% of South African youth from the eThekwini District had been vaccinated against COVID-19 by May 2022, comparable to national data where approximately 37% of young people (18–34 years) were reported to have received a COVID vaccination by mid-2022 [44].

Our study presents novel findings on vaccination behaviour among youth in South Africa, helping address a notable gap in the COVID-19 vaccination literature, which has largely focused on high-income country settings, adult populations, and health‑care settings [45–47]. Vaccine hesitancy has emerged as a significant global issue, undermining immunization efforts against vaccine‑preventable diseases [48]. Although hesitancy and uptake vary regionally, influenced by complex and interconnected factors, including social and structural determinants of health [5,37], youth represent an important population of concern, given demonstrated lower rates of vaccine uptake and higher hesitancy, often associated with lower perceived risk, lower vaccine literacy and higher misinformation [49,50].

Our results are in-line with research indicating that personal experiences, such as knowing someone who has died or has been severely impacted by the illness, have significant social and psychological impacts, including a positive association with vaccine uptake [51–54]. For example, in our study, we found that women were 5 times more likely to be vaccinated when they had personal experiences of death due to COVID-19. In global and local media, youth were largely blamed for COVID-19 transmission [55,56], and though not identified as high-risk for COVID-19 disease complications [57], young people were vulnerable to economic and social impacts, and mental‑health trauma [58–60], including COVID-related death or disability of parents, caregivers or other relatives, and friends [61]. Our results notably show large proportions of young people who personally knew individuals who had been hospitalized or who had died due to COVID-19 illness. This may be explained in part by the fact that many of our study participants lived in multi-generational homes [24], with three-quarters reporting one or more seniors in the home. Studies have found that protecting oneself or others was a strong predictor of vaccination acceptance [35], including in South African studies reporting that living with someone who is vaccinated was a strong predictor of COVID-19 vaccination status [36,38]. Given higher rates of vaccination among those 60 years of age or older [13], this may further help explain higher vaccination rates among those with older adults in the home.

An increase in age was associated with COVID-19 vaccination, where each one-year increase from the sample mean was associated with higher odds of being vaccinated. While being older is a predictor of vaccination status among adults [35], studies specific to young adults have found mixed results regarding age as a predictor of vaccination status and acceptance [62,63]. It is possible that our results may have been impacted by changes in age-group related vaccine coverage for those 18 and over, who were granted access in July 2021 just prior to the start of the study [10]. Vaccination disparities by ethnicity for men in our study are in line with South African data, which indicate that individuals of white and Asian/Indian descent are more likely to be vaccinated compared with Black and Coloured South Africans [64,65], and likely reflect both socio-economic differences, as well as differences in attitudes, perceptions, and vaccine mistrust amongst social groups [18,66]. In South Africa, ethnic and/or racial identity strongly correlates with socioeconomic status and health outcomes, as a result of deeply rooted historical oppression and the legacy of apartheid-era policies [67,68]. However, given the lack of precision for those identifying as white in our study sample, further research may be needed to explore the relationship between ethnicity and vaccine uptake.

Employment was also a significant predictor of vaccination for both men and women, although results differed in full and reduced models. In early 2021, the South African government introduced guidelines for mandatory workplace vaccine policies [69], and though this may help explain higher rates of vaccinations among some participants, vaccination mandates were never widely adopted, including in the public sector. Data suggest that South African youth were economically impacted by the pandemic, with an unemployment rate of 63.9% for 15–24 year olds in the first quarter of 2022, and 37% of youth not in any employment, education or training programs [70]. Furthermore, while COVID-19 vaccinations were provided free-of-charge through government programming [71], we hypothesize that the reported lack of timely access as well as delays in distribution [11,12] may have impacted accessibility for those in lower socioeconomic positions, including those in our study. Studies indicate that women and lower-wage workers were disproportionately impacted by unemployment in the first few years of the pandemic [72,73], which may help explain the gendered difference in the relationship between employment status and vaccination. We also found that for women, having children was associated with a 33% lower likelihood of vaccination, which may be indicative of concerns about side effects, fertility, and impacts on the fetus among women of reproductive age [74–76].

Our finding that experiencing xenophobia since the start of the pandemic is a significant predictor of vaccination status was unexpected, and differs from global data indicating lower uptake among migrants and ethnic minorities due to higher hesitancy, and greater access barriers [39]. A recent South African qualitative study [77] showed that experiences of migrants varied from exclusionary to inclusionary, suggesting that in the South African context, xenophobia may have influenced vaccination behaviour in contrasting ways, including that compliance with vaccination may serve as a practical tool for economic and social participation. It is possible that this result was also influenced by unmeasured factors, including vaccination completed in home countries of origin. Further research would be beneficial exploring the vaccination experiences and intentions of immigrants and ethnic minorities in South Africa.

We found that identifying as LGBQ+ was also a significant predictor of vaccination for both men and women in our study. While some research indicates that members of LGBQ+ communities have been disproportionately impacted by the pandemic, there is limited research examining vaccine uptake and acceptance among sexual minority individuals [78]; as such, these results should be interpreted with caution require and further investigation and exploration of unmeasured or uncontrolled factors that may influence this relationship in the South African context.

Similar to other South African youth studies on hesitancy to vaccinate [22,65], participants who were eligible but not vaccinated, expressed concerns about effectiveness and safety/side effects. We found that concerns about side-effects were higher among women, while among unvaccinated men we found higher responses to questions indicating a lack of perceived need for vaccination and perceptions of natural immunity/strength. While additional qualitative research is needed to better understand gendered hesitancy behaviours, our findings are aligned with other research showing that young women may be particularly concerned about side effects and vaccine safety, due to reproductive intentions [22,76]. Studies examining the relationship between COVID-19 and masculinity have found a negative relationship between men’s ascription to normative gender roles and COVID-19 precaution [79]. Our results point to the need to address hesitancy and misinformation among South African youth, explore potential gendered dimensions of risk, uncover distinct patterns of hesitancy determinants, and require further examination, including through qualitative inquiry.

While several of our predictors, including age, ethnicity and sexual orientation, are not directly modifiable, our findings point to more actionable opportunities for designing future youth centred public health campaigns. For example, our findings on multi-generational living, as well knowing someone who was hospitalized or died due to COVID, suggest that community-level protection may be an area for public health emphasis. In a setting like eThekwini, municipal health teams could deploy ward-based community health workers to conduct door-to-door engagements that emphasize the importance of vaccination, especially for vulnerable populations such as the elderly. Given that our findings indicate gender differences in non-vaccination reasons and concerns about safety and efficacy, tailored gender-specific misinformation education targeting youth may be more effective for addressing concerns across groups. For young women, campaigns can directly counter misinformation surrounding reproductive health, side-effects, and fertility fears by partnering with local youth-friendly clinics and trusted female clinicians. Finally, our multi-pronged recruitment approach, as well as evident engagement in online research surveys, suggest that such channels may be useful for findings dissemination and communications addressing misinformation. Moreover, to maximize the translational reach of these interventions, public health departments can utilize low-barrier digital tools like #datafree messaging platforms and collaborate with peer-led advisory groups, such as our Adolescent Community Advisory Board (aCAB) to co-design and disseminate highly interactive, myth-busting content directly to youth cell phones.

### Limitations

This study has several limitations. First, our exclusion of participants who received only one AstraZeneca vaccination reduced our study sample by 12%. However, despite this limitation, we believe that this approach reflected a more accurate representation of confirmed vaccination within our study sample and increased the specificity of our outcome measure. AstraZeneca was never granted full approval for public use in South Africa, and was provided only as part of a limited, clinical trial aimed at assessing effectiveness against current COVID-19 variants [32–34]. Given the limited circumstances under which AstraZeneca was available in South Africa, individuals who received AstraZeneca could not have received it through the national program, and our survey did not capture information on the specific circumstances or their reasons for receiving only one dose, including how findings of low effectiveness of the vaccination may have impacted future vaccination behaviour [33]. As such, we felt it best to exclude these individuals from the study sample and note that individuals who received only one dose of AstraZeneca may have differed systematically from the rest of the study sample, given reported differences between vaccine trial participants and the general population [80]. We do, however, acknowledge that excluding these individuals may have introduced bias, limiting our generalizability.

The cross-sectional nature of the data limits causal inference, and we note that unmeasured factors may also limit the generalizability of our results. In particular, we cannot rule out the possibility of residual confounding from unmeasured variables that were unavailable for inclusion in our analyses such as political attitudes, trust in the healthcare system, or historical vaccination behaviors, all of which are known to influence vaccine acceptance and uptake. Furthermore, we acknowledge that stepwise selection, despite being a commonly used statistical method, has known limitations, including reduced generalizability and replicability. As such, although we present both full and reduced model results, we prioritized the full model as our primary analysis in the findings reporting. Convenience sampling, via an online survey, is also a potential source of bias given our recruitment approach and may limit the generalizability of our findings beyond the sample population. The exclusion of individuals who completed the survey in less than 4.5 min may bias the results. However, given the median survey completion time of 15 min [31], the research team determined that 4.5 min was insufficient time to adequately complete the questionnaire. Due to the low number (*n* = 52) of participants identifying as non-binary or gender non-conforming, low cell counts prevented gender-stratified prediction analysis for this group. Future research with adequately powered samples would be beneficial to help identify vaccination predictors specific to non-binary and gender non-conforming individuals. As vaccination status was self-reported, social desirability bias is a concern, and we cannot rule out under‑ or over-reporting of actual vaccination status. Although Moderna was included as a vaccine response option, it was available only through the Sisonke trial for healthcare workers [81].

## Conclusion

We found that just under 38% of South African youth in our study had been vaccinated against COVID-19, with the strongest predictors of COVID vaccination including having seniors in the home and knowing individuals who were hospitalized or had passed away due to COVID-19, as well as experiences of xenophobia during the COVID-19. Understanding why vaccination rates may be higher among certain populations (e.g. LGBQ+ individuals, those experiencing xenophobia) requires further investigation. Given the low rates of vaccination and high rates of vaccine hesitancy related to safety and effectiveness concerns, future research could focus on better understanding modifiable factors to support vaccine uptake, including differences across social groups, as well as focusing on better understanding drivers of vaccine hesitancy related to mistrust and misinformation. Such evidence will be central to supporting South Africa’s immunization programs among youth, as well as to more targeted, demographically appropriate public health communication messaging in preparation for future pandemics and other vaccination campaigns.

## Supplementary Material

AYAZAZI Rights_Supplementary Materials.docx

STROBE Checklist.docx

## Data Availability

Due to the sensitive nature of the questions asked in this study, data cannot be shared publicly. Requests to access the data should be directed to Dr. Angela Kaida, angela_kaida@sfu.ca.
