# Supplementary material for: Predictors of COVID-19 vaccination status among youth in eThekwini, South Africa: results from the AYAZAZI RIGHTS study
Source: Glob Health Action. 2026 Oct 6;19(1):2737555. doi: 10.1080/16549716.2026.2737555 (PMC13644445; doi:10.1080/16549716.2026.2737555)
Supplement: Supplementary file 2 [file ZGHA_A_2737555_SM2220.docx]

**Supplementary Table A: Vaccine status and COVID-19 related variables among participants (n=1383)**

|  |  | **Overall (n=1383)** |
| --- | --- | --- |
| **Have you received a COVID-19 vaccination?** | no | 859 (62.1) |
|  | yes | 524 (37.9) |
| **Ever had COVID-19?** | no | 832 (60.2) |
|  | yes | 550 (39.8) |
| **Vaccination status** | fully vaccinated | 328 (23.7) |
|  | partially vaccinated | 196 (14.2) |
|  | not vaccinated | 859 (62.1) |
| **If a COVID-19 vaccine was available to you, how likely are you to receive it?** | already vaccinated | 524 (37.9) |
|  | very likely | 82 (5.9) |
|  | somewhat likely | 81 (5.9) |
|  | neutral | 218 (15.8) |
|  | unlikely | 288 (20.8) |
|  | very unlikely | 190 (13.7) |
| **How important is it to you to get the COVID-19 vaccine to protect the health of your community?** | extremely | 365 (26.6) |
|  | very | 268 (19.5) |
|  | moderately | 225 (16.4) |
|  | a little | 260 (18.9) |
|  | not at all | 255 (18.6) |
| **How important is it to you to get the COVID-19 vaccine to protect your own health?** | extremely | 329 (23.8) |
|  | very | 288 (20.9) |
|  | moderately | 216 (15.7) |
|  | a little | 258 (18.7) |
|  | not at all | 289 (20.9) |

**Supplementary Table B: Factors predicting vaccination status among AYAZAZI Rights study participants ≥18 with interactions, by gender (n=1331)**

|  |  | **Young Men (n=585)** | | | | | | **Young Women (n=746)** | |
| --- | --- | --- | --- | --- | --- | --- | --- | --- | --- |
|  |  | **Model 3 Income*xenophobia** | | **Model 4 student*xenophobia** | | **Model 5**  **Age*seniors in home** | | **Model 6**  **Age*seniors in home** | |
|  |  | aOR | 95% CI | aOR | 95% CI | aOR | 95% CI | aOR | 95% CI |
| Intercept |  | 0.05 | 0.01 – 0.19 | 0.06 | 0.02 – 0.22 | 0.07 | 0.02 – 0.24 | 0.08 | 0.02 – 0.23 |
| Centered age (mean) |  | 1.25 | 1.09 – 1.44 | 1.31 | 1.14 – 1.51 | - | - | - | - |
| Ethnicity | Black | REF |  | REF |  | REF |  | REF |  |
|  | Coloured | 0.47 | 0.20 – 1.10 | 0.48 | 0.21 – 1.11 | 0.48 | 0.21 – 1.11 | 1.10 | 0.62 – 1.94 |
|  | Indian/Asian | 2.52 | 1.11 – 5.71 | 2.29 | 1.01 – 5.20 | 2.52 | 1.10 – 5.79 | 1.39 | 0.76 – 2.55 |
|  | white | 12.56 | 3.75 – 42.04 | 11.94 | 3.59 – 39.65 | 11.90 | 3.59 – 39.53 | 0.78 | 0.32 – 1.89 |
| Sexual Orientation | heterosexual | REF |  | REF |  | REF |  | REF |  |
|  | LGBQ+ | 2.40 | 1.09 – 5.29 | 2.35 | 1.06 – 5.20 | 2.44 | 1.10 – 5.40 | 2.28 | 1.31 – 3.99 |
| Currently a student | no | REF |  | - |  |  |  |  |  |
|  | yes | 0.90 | 0.52 – 1.57 | - | - | 0.94 | 0.54 – 1.62 | 1.00 | 0.67 – 1.49 |
| Currently Employed | no | REF |  | REF |  | REF |  | REF |  |
|  | yes | 2.36 | 1.12 – 4.97 | 2.30 | 1.09 – 4.82 | 2.27 | 1.08 – 4.79 | 1.42 | 0.83 – 2.42 |
| Relationship Status | single | REF |  | REF |  | REF |  | REF |  |
|  | cohabitating | 0.77 | 0.26 – 2.30 | 0.80 | 0.26 – 2.44 | 0.82 | 0.28 – 2.43 | 2.48 | 1.17 – 5.27 |
|  | not cohabitating | 0.40 | 0.20 – 0.81 | 0.37 | 0.19 – 0.75 | 0.37 | 0.19 – 0.75 | 0.66 | 0.39 – 1.11 |
| Have children | no | REF |  | REF |  | REF |  | REF |  |
|  | yes | 1.25 | 0.71 – 2.18 | 1.15 | 0.66 – 2.02 | 1.20 | 0.68 – 2.09 | 0.67 | 0.45 – 1.02 |
| Income level | R0-R1600 | - |  | REF |  | REF |  | REF |  |
|  | R1601+ | - | - | 1.00 | 0.51 – 1.96 | 1.05 | 0.53 – 2.05 | 1.25 | 0.78 – 2.02 |
| Number of children in household | none | REF |  | REF |  | REF |  | REF |  |
|  | one to two | 1.95 | 0.82 – 4.62 | 2.23 | 0.93 – 5.33 | 2.04 | 0.85 – 4.89 | 1.15 | 0.54 – 2.45 |
|  | three or more | 1.72 | 0.70 – 4.22 | 1.78 | 0.72 – 4.40 | 1.87 | 0.76 – 4.64 | 1.35 | 0.60 – 3.04 |
| Number of seniors in household | none | REF |  | REF |  | - |  | - |  |
|  | one | 1.13 | 0.63 – 2.02 | 1.07 | 0.60 – 1.92 | - | - | - | - |
|  | two or more | 4.49 | 2.19 – 9.20 | 3.89 | 1.90 – 7.98 | - | - | - | - |
| Known people hospitalized due to COVID-19 | none | REF |  | REF |  | REF |  | REF |  |
|  | one to five | 0.81 | 0.36 – 1.83 | 0.76 | 0.34 – 1.73 | 0.75 | 0.34 – 1.68 | 1.20 | 0.58 – 2.51 |
|  | five or more | 2.67 | 1.09 – 6.54 | 2.41 | 0.98 – 5.94 | 2.72 | 1.12 – 6.61 | 3.54 | 1.57 – 7.97 |
| Known people died due to COVID-19 | none | REF |  | REF |  | REF |  | REF |  |
|  | one to five | 2.50 | 1.27 – 4.93 | 2.25 | 1.14 – 4.44 | 2.13 | 1.11 – 4.09 | 1.88 | 1.08 – 3.27 |
|  | five or more | 14.04 | 6.37 – 30.94 | 13.36 | 6.08 – 29.36 | 12.06 | 5.59 – 26.03 | 4.94 | 2.64 – 9.24 |
| HIV Status | negative | REF |  | REF |  | REF |  | REF |  |
|  | positive | 0.72 | 0.21 – 2.42 | 0.65 | 0.19 – 2.25 | 0.63 | 0.19 – 2.12 | 0.90 | 0.49 – 1.68 |
|  | unknown/PTNA | 0.84 | 0.51 – 1.38 | 0.88 | 0.53 – 1.45 | 0.81 | 0.49 – 1.33 | 0.90 | 0.59 – 1.39 |
| Experienced xenophobia since start of | no | - |  | - |  | REF |  | REF |  |
| Pandemic | yes | - | - | - | - | 6.43 | 2.89 – 14.32 | 3.86 | 1.84 – 8.10 |
| Income(R0-R1600)*xenophobia |  | 23.20 | 6.26 – 86.03* |  |  |  |  |  |  |
| Income(R1600+)*xenophobia |  | 2.96 | 0.03 – 0.59* |  |  |  |  |  |  |
| Student*xenophobia (no) |  |  |  | 16.58 | 5.38 – 51.13* |  |  |  |  |
| Student*xenophobia (yes) |  |  |  | 1.37 | 0.38 – 5.03* |  |  |  |  |
| Centered age*seniors in home (none) |  |  |  |  |  | 2.23 | 0.97 – 1.45 | 1.07 | 0.89 – 8.10 |
| Centred age*seniors in home (one) |  |  |  |  | - | 1.19 | 0.99– 1.43 | 1.35 | 1.10 – 1.64* |
| Centred age *seniors in home (two or more) |  |  |  |  | - | 1.55 | 1.20 – 2.01* | 1.43 | 1.23 – 1.66* |
| **Model performance** |  |  | |  | |  | |  | |
| Observations (n) |  | 585 | | 585 | | 585 | | 746 | |
| R-squared |  | 0.464 | | 0.471 | | 0.459 | | 0.321 | |
| c-statistic |  | 0.889 | | 0.889 | | 0.889 | | 0.824 | |
| Hos-Lemeshow test (p-value) |  | 0.921 | | 0.470 | | 0.353 | | 0.007 | |
| aOR: adjusted odds ratio, 95% CI: 95% confidence interval; m(SD): mean and standard deviation; c-stat: concordance statistic; COVID-19: Coronavirus disease 2019; LGBQ+: lesbian, gay, bisexual, queer, and more; HIV: Human Immunodeficiency virus; PTNA: prefer not to say  *p<0.05 | | | | | | | | | |

**Supplementary Table C: Generalized Variance Inflation Factor(s) for Candidate Predictors**

| **Variable** | **GVIF** | **Df** | **GVIF^(1/(2×Df))** |
| --- | --- | --- | --- |
| Age | 1.479 | 1 | 1.216 |
| Gender | 1.395 | 2 | 1.087 |
| Race/ethnicity | 1.274 | 3 | 1.041 |
| Sexual orientation | 1.385 | 1 | 1.177 |
| Monthly income | 3.463 | 4 | 1.168 |
| Current Student | 1.355 | 1 | 1.164 |
| Employed | 2.661 | 1 | 1.631 |
| Relationship status | 1.467 | 2 | 1.101 |
| Have children | 1.374 | 1 | 1.172 |
| Number of children in household | 1.370 | 2 | 1.082 |
| Number of seniors in household | 1.455 | 2 | 1.098 |
| Known people hospitalized from COVID-19 | 1.309 | 2 | 1.070 |
| Known people who died from COVID-19 | 1.442 | 2 | 1.096 |
| HIV status | 1.189 | 2 | 1.044 |
| Experienced xenophobia since start of Pandemic | 1.209 | 1 | 1.100 |
| *GVIF = generalized variance inflation factor; Df = degrees of freedom* | | | |
